# Nuclear factor 90 promotes angiogenesis by regulating HIF-1α/VEGF-A expression through the PI3K/Akt signaling pathway in human cervical cancer

**DOI:** 10.1038/s41419-018-0334-2

**Published:** 2018-02-15

**Authors:** Wenqian Zhang, Zhengai Xiong, Tianqin Wei, Qiumeng Li, Ying Tan, Li Ling, Xiushan Feng

**Affiliations:** 1 Department of Obstetrics and Gynecology, The Second Affiliated Hospital, Chongqing Medical University, 400010 Chongqing, China; 2 Department of Neurosurgery, The Second Affiliated Hospital, Chongqing Medical University, 400010 Chongqing, China

## Abstract

Vascular endothelial growth factor A (VEGF-A), a fundamental component of angiogenesis, provides nutrients and oxygen to solid tumors, and enhances tumor cell survival, invasion, and migration. Nuclear factor 90 (NF90), a double-stranded RNA-binding protein, is strongly expressed in several human cancers, promotes tumor growth by reducing apoptosis, and increasing cell cycle process. The mechanisms by which cervical cancer cells inducing VEGF-A expression and angiogenesis upon NF90 upregulation remain to be fully established. We demonstrated that NF90 is upregulated in human cervical cancer specimens and the expression of NF90 is paralleled with that of VEGF-A under hypoxia. The expressions of hypoxia inducible factor-1α (HIF-1α) and VEGF-A are downregulated upon NF90 knockdown, which can be rescued by ectopic expression of NF90. Suppression of NF90 decreases the tube formation and cell migration of HUVECs. Moreover, the PI3K/Akt signaling pathway participates in the regulation. Knockdown of NF90 also reduces the tumor growth and angiogenesis of cervical cancer cell line in the mouse xenograft model. Taken together, suppression of NF90 in cervical cancer cell lines can decrease VEGF-A expression, inhibit angiogenesis, and reduce tumorigenic capacity in vivo.

## Introduction

NF90 (also known as DRBP76), originally identified as a post-transcriptional regulator of interleukin-2 (IL-2) promoter^1^, is conserved in vertebrates, and is one of the major products of alternative splicing of the interleukin enhancer-binding factor-3 (ILF3) gene^2^. NF110 (also known as ILF3), an alternative splice form of ILF3, has a distinct N-terminal with NF90, and overexpressed in malignant nasopharyngeal carcinoma cells^3^. NF90 forms a heterodimeric complex with nuclear factor 45 (NF45), a product of interleukin enhancer-binding factor-2 (ILF2) gene^4^. Further work revealed that NF90/NF45 complex participates in DNA metabolism^5,6^, transcription^7–10^, translation^11–13^, RNA export^14,15^, mRNA stability^16–18^, pri-miRNA processing^19^, replicationand gene expression of many viruses^20–26^. In contrast, NF110 is predominantly restricted to the nucleus with minor effects on the cell growth when it is reduced.

Repression of either NF90 or NF45, but not NF110, leads to the retardation of cervical cancer cell growth and the formation of giant multinucleated cells^4^. ILF3 maintains sustained uPA expression in the breast cancer cells and promotes breast tumorigenicity^27^. NF90 bounds to the 3′untranslated regions (3'-UTRs) of cyclin E1 mRNA, depletion of NF90 delays cell-cycle progression, inhibits cell proliferation, reduces tumorigenic capacity, and sensitizes hepatocellular carcinoma to the CDK (cyclin-dependent kinase) inhibitor roscovitine^18^. Previously, researchers found that the DPBP76/NF90 isoform facilitates vascular endothelial growth factor (VEGF) expression through stabilizing VEGF mRNA under hypoxia conditions, promotes the breast cancer angiogenesis in vivo and tumor progress^16^. However, the underlying regulatory mechanism by which NF90 induces upregulation of VEGF-A expression and enhancement of tumor angiogenic activity remains unclear.

The transcription factor hypoxia inducible factor-1 (HIF-1), a heterodimeric protein composed of the hypoxia-inducible α subunit and the constitutively expressed β subunit^28^, exerts a pivotal role in tumor angiogenesis. In tumor angiogenesis, only HIF-1α is tightly regulated by low-oxygen tension. HIF-1α plays an important role in cancer cell survival and progression through inducing tumor cells more aggressive to better adapt to the hypoxia of solid tumors^29,30^. It has been shown that HIF-1α is a leading regulator of tumor angiogenesis following hypoxia, because it regulates the expression of several pro-angiogenic factors, such as the VEGF^31–33^. VEGF-A is the most important component of angiogenesis, tumor growth, and metastasis^34–38^, including cervical cancer^39,40^.

In the present study, we aimed to evaluate the potential involvement of NF90 in the expression of HIF-1α/VEGF-A in cervical cancer cells and regulation of angiogenesis. To this end, we demonstrate that NF90 is upregulated in cervical cancer specimens, mediates the upregulation of HIF-1α/VEGF-A, as well as the endothelial tube formation through PI3K/Akt signaling pathway. Knockdown of NF90 in HeLa and SiHa cell lines inhibits angiogenesis and reduces the tumorigenic capacity in vivo. These results suggest an important new role of NF90 in cervical cancer angiogenesis and raise the possibility that NF90 may be a new anti-angiogenesis therapeutic target for cervical cancer.

## Results

### NF90 and NF110 are overexpressed in human cervical cancer specimens

**Fig. 1:**
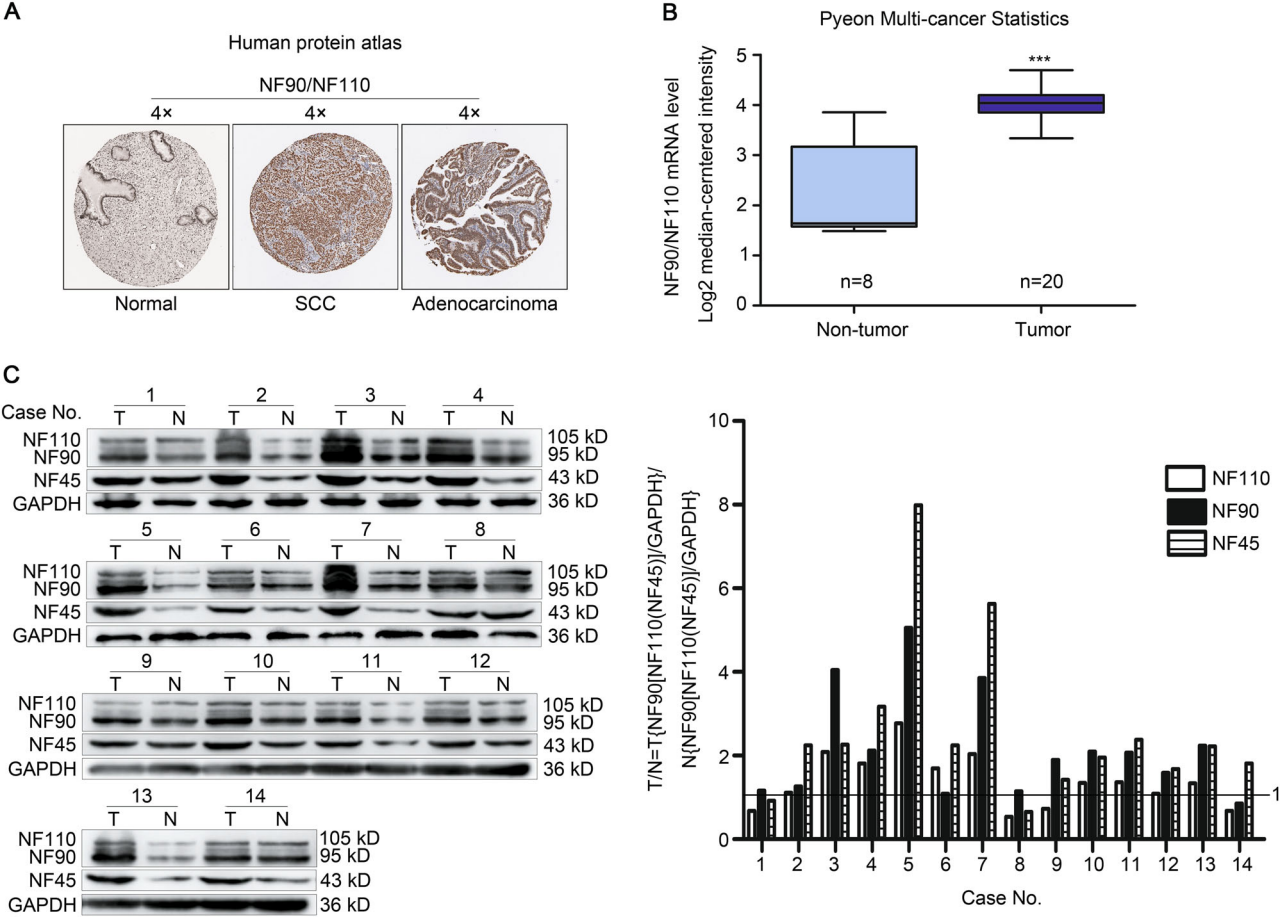
NF90 and NF110 are upregulated in human cervical cancer specimens. **a** NF90 and NF110 expression in normal cervical tissue, SCC, and adenocarcinoma specimens. Images were taken from the Human Protein Atlas (www.proteinatlas.org) online database. SCC cervical squamous cell carcinoma. **b** The mRNA expression of NF90 and NF110 in normal tissues vs. cervical cancer from the Oncomine data (www.oncomine.org) (*n* = 28), ***P < 0.001. **c** The protein levels of NF90 and NF110 in paired human cervical cancer and adjacent noncancerous cervical epithelial tissues. GAPDH was used as the loading control. The quantifications of immunoblots are shown in the right panel. T tumor, N non-tumor.

To provide initial insight into the clinical relevance of NF90 and NF110 expression, their protein expressions in clinical specimens are first analyzed from the Human Protein Atlas (www.proteinatlas.org). NF90 and NF110 had the strong positive expressions in cervical squamous cell carcinoma and adenocarcinoma, and weak negative expression in normal cervical tissues (Fig. 1a). Consistently, the mRNA level of NF90 and NF110 was higher in cervical cancer tissues than that in normal cervical tissues (4.065 ± 0.076 vs. 2.157 ± 0.345, *P *< 0.001, *n* = 28) in Pyeon Multi-cancer database (www.oncomine.org) (Fig. 1b). We also investigated the protein levels of NF90, NF110, and NF45 in 14 paired cervical cancer tissues and adjacent non-cancerous cervical epithelial tissues with western blotting. More than 85% (12 of 14) cases exhibited higher protein expressions of NF90 and NF45 in the tumors compared with their corresponding controls (Fig. 1c). Similar result was also obtained in the protein expression of NF110 (64%, 9 of 14). Thus, NF90 and NF110 are upregulated in the majority of cervical cancer in comparison with adjacent non-cancerous cervical epithelial tissues.

### NF90 is involved in the expression of VEGF-A induced by hypoxia in cervical cancer

**Fig. 2:**
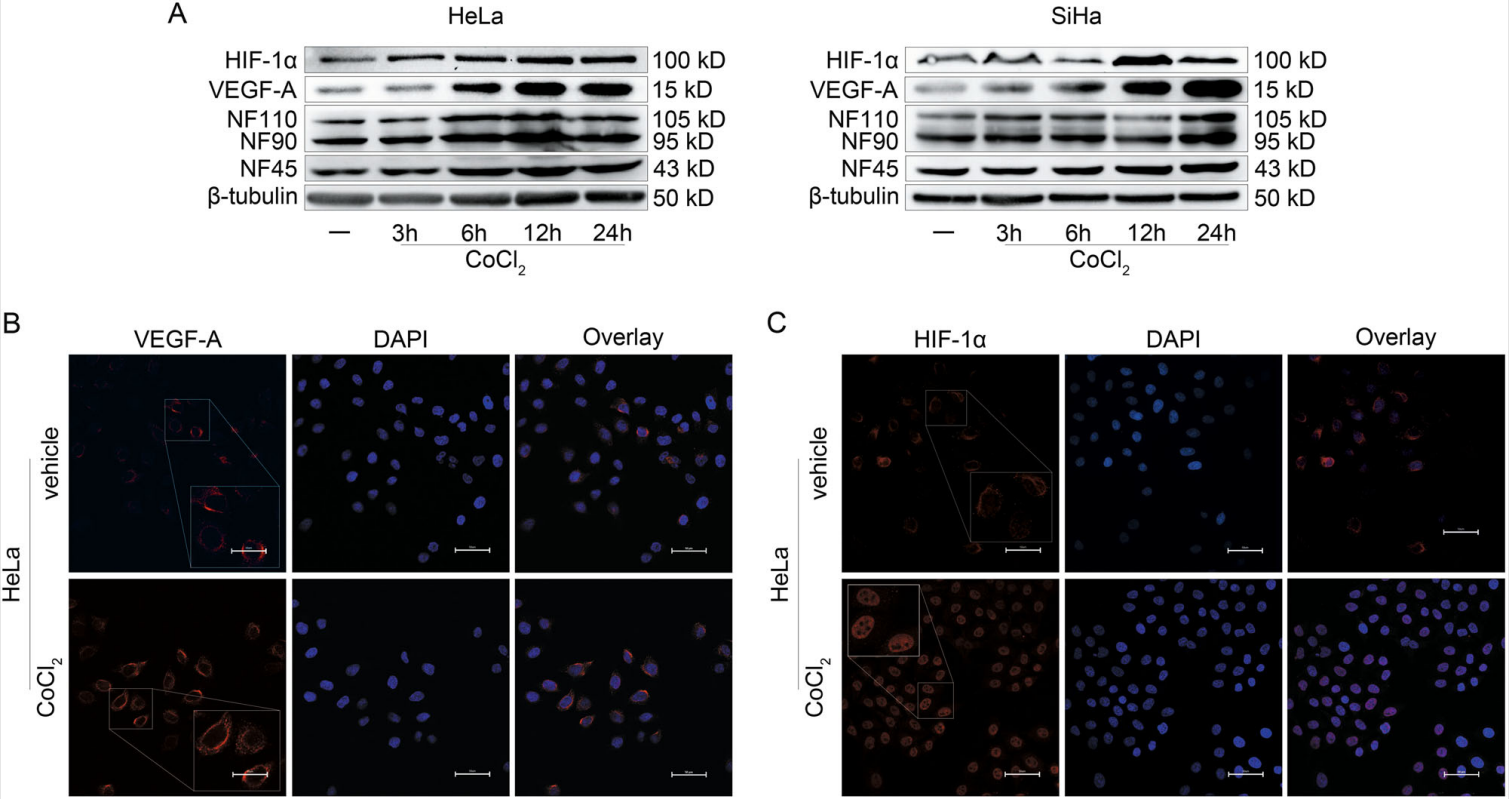
CoCl_2_ induces the expression of HIF-1α, VEGF-A, and NF90 in cervical cancer cells in a time-dependent manner. **a** After being starved overnight, cervical cancer cells were cultured with CoCl_2_ (150 μM) up to 24 h. The expressions of HIF-1α, VEGF-A, NF90, NF45, and NF110 were determined by western blotting. **b** HeLa cells were seeded in a 24-well plate and starved overnight, then CoCl_2_ (150 μM) was applied for 12 h. The VEGF-A protein expression was evidenced by confocal microscopy. Red signal, VEGF-A; blue signal, nuclei labeled with DAPI. Magnification, ×400; scale bars = 50 µm. Images are shown representative of 10 random fields of 3 independent experiments. **c** The HIF-1α protein expression induced by CoCl_2_ was evidenced by confocal microscopy. Red signal, HIF-1α; blue signal, nuclei labeled with DAPI. Magnification, ×400; scale bars = 50 µm. Images are shown representative of 10 random fields of 3 independent experiments.

To evaluate whether hypoxia may promote the expression of HIF-1α and its important target gene VEGF-A in cervical cancer. Human cervical cancer cell lines (HeLa and SiHa) were treated from 3 to 24 h with the hypoxia mimetic agent Cobalt (II) chloride hexahydrate (CoCl_2_) and a low-oxygen tension (2% O_2_). The protein expressions of HIF-1α, VEGF-A, and NF90 were induced by CoCl_2_ and low-oxygen tension (2% O_2_) in a time-dependent manner in both HeLa and SiHa cell lines (Fig. 2a, Supplementary Fig. 1). In particular, the increased protein expressions of NF90 and NF45 were paralleled with that of VEGF-A both in HeLa and SiHa cells. While, there was no difference in the protein expression of NF110 induced by CoCl_2_ or low-oxygen tension. CoCl_2_ and low-oxygen tension had similar effect of hypoxia induction, and we chose CoCl_2_ incubated with HeLa cell lines for 12 h and with SiHa cell lines for 24 h in the following studies, respectively.

The protein expressions of HIF-1α and VEGF-A induced by CoCl_2_ in HeLa cell lines were also evidenced by confocal microscopy. The protein expression of VEGF-A in cytoplasm was elevated by CoCl_2_ stimulation, observed by red signal (Fig. 2b). The CoCl_2_ stimulation increased and transferred the cytoplasmic expression of HIF-1α to nuclear (Fig. 2c).

### Knockdown of NF90 decreases HIF-1α/VEGF-A protein expressions in cervical cancer cell lines

**Fig. 3:**
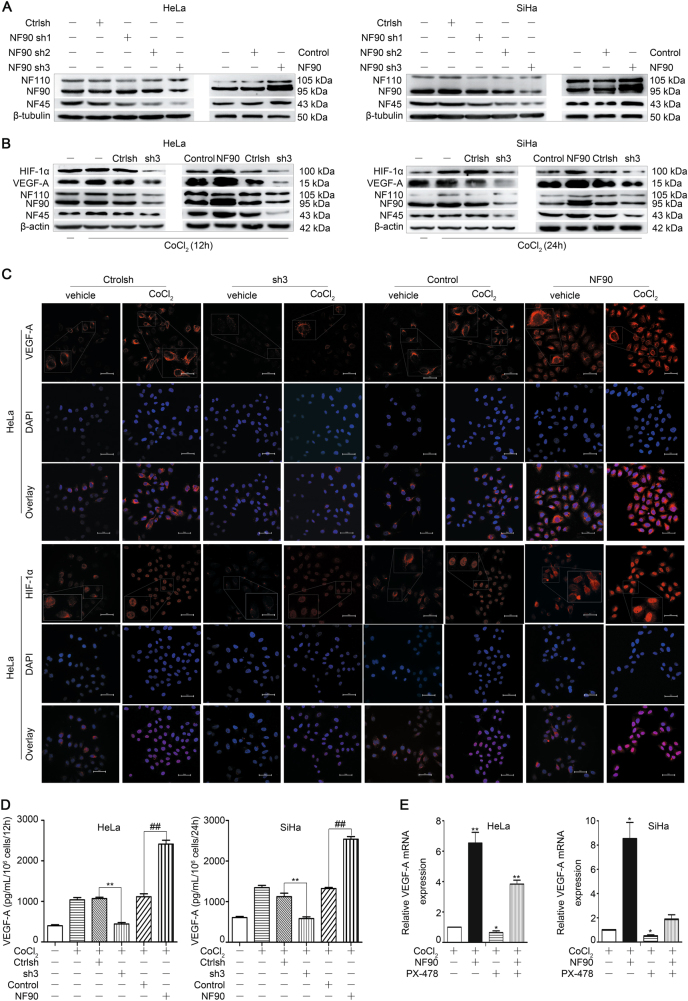
Knockdown of NF90 decreases VEGF-A expression. Cervical cancer cells were seeded in six-well plates overnight and then transfected with NF90 shRNA (sh1-3) or shRNA control (ctrolsh) for knockdown, and NF90 or overexpression control (control) for overexpression. **a** The knockdown and overexpression of NF90 in cervical cancer cells were confirmed by western blotting. **b** Control, NF90, ctrolsh or sh3 cells were stimulated with CoCl_2_ (150 µM) for indicated times and then subjected to western blotting to detect the expressions of HIF-1α, VEGF-A, NF110, NF90, NF45, and β-actin proteins. **c** Evaluation of VEGF-A and HIF-1α expressions by confocal microscopy. After seeded in 24-well plates and starved overnight, HeLa cells, which had been transfected with control, NF90, ctrolsh or sh3, were stimulated for 12 h with vehicle or CoCl_2_ (150 µM), as indicated. Red signal on the upper line, VEGF-A; red signal on the lower line, HIF-1α; blue signal, nuclei stained by DAPI. Magnification, ×400; scale bars = 50 µm. Images are shown representative of 10 random fields of 3 independent experiments. **d** The VEGF-A levels in cell supernatants were evaluated using human VEGF-A ELISA kit following the manufacturer’s instructions. Column, mean (*n* = 3); bars, SD. **P < 0.01, compared with ctrolsh conditioned medium. ^##^*P* < 0.01, compared with control-conditioned medium. **e** Stable transfected HeLa and SiHa cells were cultured with CoCl_2_ for indicated times after pre-treated for 30 min with HIF-1α inhibitor PX-478. The VEGF-A mRNA expression was confirmed by qRT-PCR. *P < 0.05, **P < 0.01. All experiments were repeated in triplicate.

To confirm whether NF90 may increase VEGF-A-dependent angiogenesis in human cervical cancer cell lines, the stable NF90 knockdown and overexpression cervical cancer cells were established through lentiviral transfection. As NF90 and the larger alternative splice variant NF110 might have distinct functions, the protein expression of NF110 has also been observed during the following experiments. qRT-PCR and western blotting showed that the NF90 shRNA3 (sh3) selectively represses the expression of NF90 mRNA (Supplementary Fig. 2) and protein (Fig. 3a). In addition, the mRNA and protein expression of NF45 was partially depleted in NF90 shRNA3 transfected cells. This co-regulation is owing to the mutual stabilization of the proteins in the NF90/NF45 heterodimeric core complex^4,41^. On the other hand, the mRNA or protein expression of NF110 was not influenced by NF90 shRNA3.

The induction of VEGF-A and HIF-1α protein expression levels stimulated with CoCl_2_ were abolished by NF90 depletion, and upregulated by NF90 overexpression, as evidenced by western blotting and confocal microscopy (Fig. 3b, c), highlights the involvement of NF90 in the regulation of VEGF-A induced by hypoxia. Since VEGF-A is a secretory protein, the supernatants of knockdown and overexpression cells were collected, centrifuged, and evaluated using human VEGF-A ELISA kit following the manufacturer’s instructions. The concentration of VEGF-A was reduced in NF90 shRNA3-conditioned medium (CM), and increased in NF90 overexpression CM (Fig. 3d). Moreover, HIF-1α inhibitor PX-478 was used to show the direct involvement of HIF-1α in VEGF-A increment under hypoxia. The VEGF-A mRNA level increased by NF90 overexpression was recovered by PX-478 (Fig. 3e). Taken together, these results suggested that NF90 is involved in the regulation of VEGF-A expression induced by hypoxia through HIF-1α-dependent way.

### Downregulation of NF90 inhibits angiogenesis in vitro

**Fig. 4:**
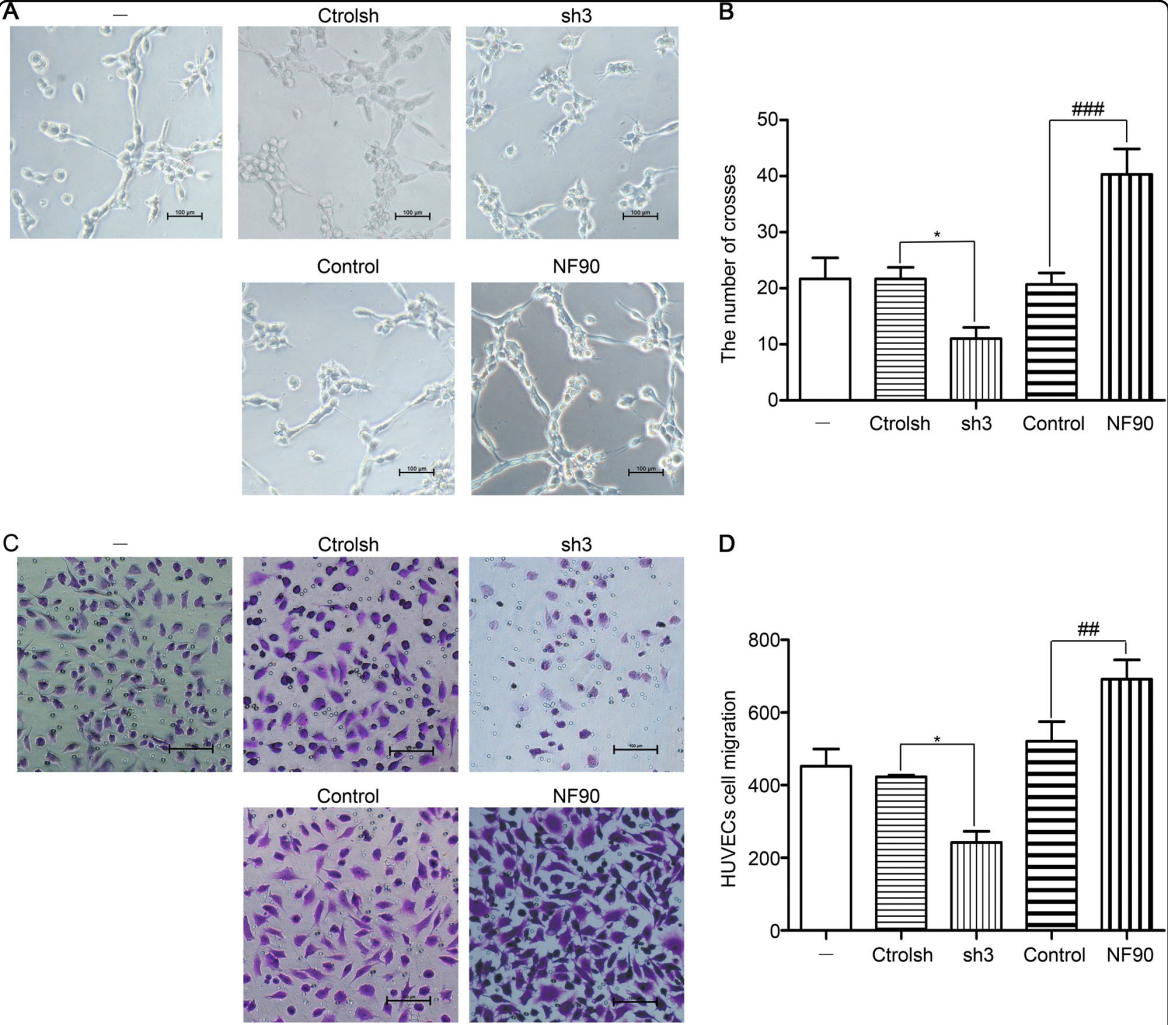
Depletion of NF90 decreases angiogenesis in vitro. Conditioned medium (CM) were collected from HeLa infected with control shRNA (ctrolsh), NF90 shRNA3 (sh3), overexpression control (control), or NF90. After being starved overnight, cells were stimulated with CoCl_2_ (150 µM) for 12 h. **a** Tube formation was evaluated in HUVECs cultured in CM. Magnification, ×100; scale bars = 100 µm. All experiments were repeated in triplicate. **b** The tube crosses in **a** data were evaluated by Image-Pro Plus 6.0, and analyzed using one-way ANOVA. Column, mean (*n* = 3); bars, SD. *P < 0.05, compared with ctrolsh; ^###^*P*<0.001, compared with control. Images shown are representative of five random fields of three independent experiments. **c** A total of 10,000 HUVECs were seeded in the upper chamber of Transwell, incubated in conditioned media for 24 h, fixed with 4% formaldehyde and stained with 0.1% crystal violet. The migrated cells were observed by microscope (×100), scale bars = 100 µm. Images shown are representative of five random fields of three independent experiments. **d** The migrated cells were counted with Image-Pro. Plus 6.0. Results were analyzed using one-way ANOVA. Column, mean (*n* = 3); bars, SD. *P < 0.05, compared with ctrolsh. ^##^*P* < 0.01, compared with control.

The formation of tubule-like structures represents a useful model system for the evaluation of the neo-angiogenesis process^42^. VEGF-A, a critical component of tumor angiogenesis, growth, and metastasis^34–38^, is regulated by NF90. To evaluate the potential role of NF90 in angiogenesis, we then collected CM from lentivirus-infected and then CoCl_2_-stimulated HeLa cells and used as culture media for human umbilical vein endothelial cells (HUVEC)s. HUVECs were plated on Matrigel, and tube/capillary-like structure formation was examined. Notably, HUVECs cultured in CoCl_2_ (150 µM) medium from HeLa, ctrolsh or control assembled into cord-like structures, while the tube formation of NF90 sh3 was no longer observed. Moreover, HUVECs grown in medium from NF90 maintained CoCl_2_ (150 µM) stimulation and displayed a complex ramified network of tubules (Fig. 4a). Compared with the ctrolsh, the number of crosses was markedly decreased in NF90 sh3 cells, while overexpression of NF90 increased the tube formation (Fig. 4b). Additionally, knockdown of NF90 significantly suppressed CM-mediated HUVECs migration, while, overexpression promoted it (Fig. 4c, d). Collectively, these results implied that NF90 upregulates VEGF-A expression, promotes the vascular tube formation and migration by HUVECs, which may contribute to angiogenesis in vitro.

### NF90 regulates VEGF-A expression through PI3K/Akt signaling

**Fig. 5:**
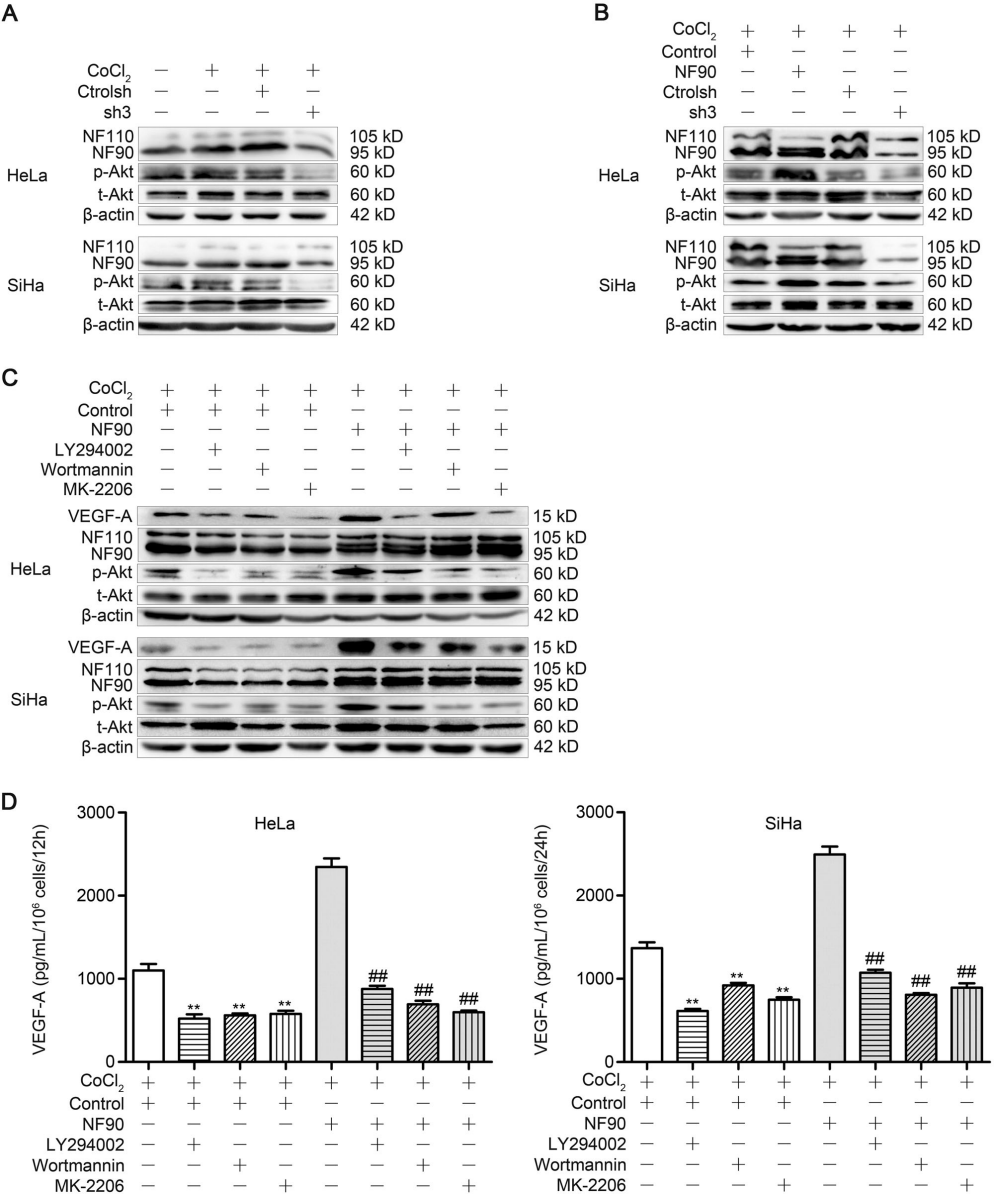
PI3K/Akt signaling participates in NF90-increased VEGF-A expression. **a** Cervical cancer cells were transfected with NF90 shRNA control (ctrolsh) or NF90 shRNA3 (sh3), then stimulated with CoCl_2_ (150 µM) for indicated durations. Phosphorylation Akt (p-Akt), total Akt (t-Akt), NF110, and NF90 were confirmed by western blotting. **b** Stable transfected cervical cancer cells were stimulated with CoCl_2_ for indicated times, p-Akt, t-Akt, NF110, and NF90 were confirmed by western blotting. **c** Stable transfected cervical cancer cells were pre-treated for 30 min with PI3K inhibitor, LY294002 (50 µM), Wortmannin (1 µM), and Akt inhibitor MK-2206 (20 nM). Then cultured with CoCl_2_ (150 µM) for indicated times. VEGF-A, p-Akt, t-Akt, NF110, and NF90 were confirmed by western blotting. **d** VEGF-A protein expression of conditioned medium was determined by  ELISA in cervical cancer cells. The results were analyzed using one-way ANOVA. Column, mean (*n* = 3); bars, SD. **P < 0.01, compared with control. ^##^*P* < 0.01, compared with NF90. All experiments were repeated in triplicate

PI3K/Akt signaling pathway has been found to participate in the regulation of many cancer-related genes, including VEGF^43–45^. We examined whether PI3K/Akt pathway is involved in the NF90-increased VEGF-A expression and angiogenesis. Knockdown of NF90 markedly inhibited the p-Akt/t-Akt ratio (Fig. 5a). In contrast, NF90 overexpression significantly upregulated Akt phosphorylation (Fig. 5b), these results suggested the activation of PI3K/Akt pathway by NF90 expression. To explore the mechanisms that may explain how NF90 affect PI3K/Akt signaling, different pharmacological inhibitors were applied. The stable overexpression control or NF90 overexpression cervical cancer cell lines was stimulated with CoCl_2_ for indicated times after pre-treated for 30 min with PI3K inhibitor (LY294002, Wortmannin) and Akt inhibitor (MK-2206). And we found that the NF90-induced VEGF-A expression and Akt phosphorylation were abolished by PI3K and Akt inhibitors, without the change of NF90 or NF110 protein expressions (Fig. 5c). The VEGF-A concentration of CM further corroborated the participation of PI3K/Akt pathway (Fig. 5d). Based on these results, it appears that NF90 acts through the PI3K/Akt pathway to enhance VEGF-A expression in human cervical cancer cell lines.

### Knockdown of NF90 decreases angiogenesis and tumor growth in vivo

**Fig. 6:**
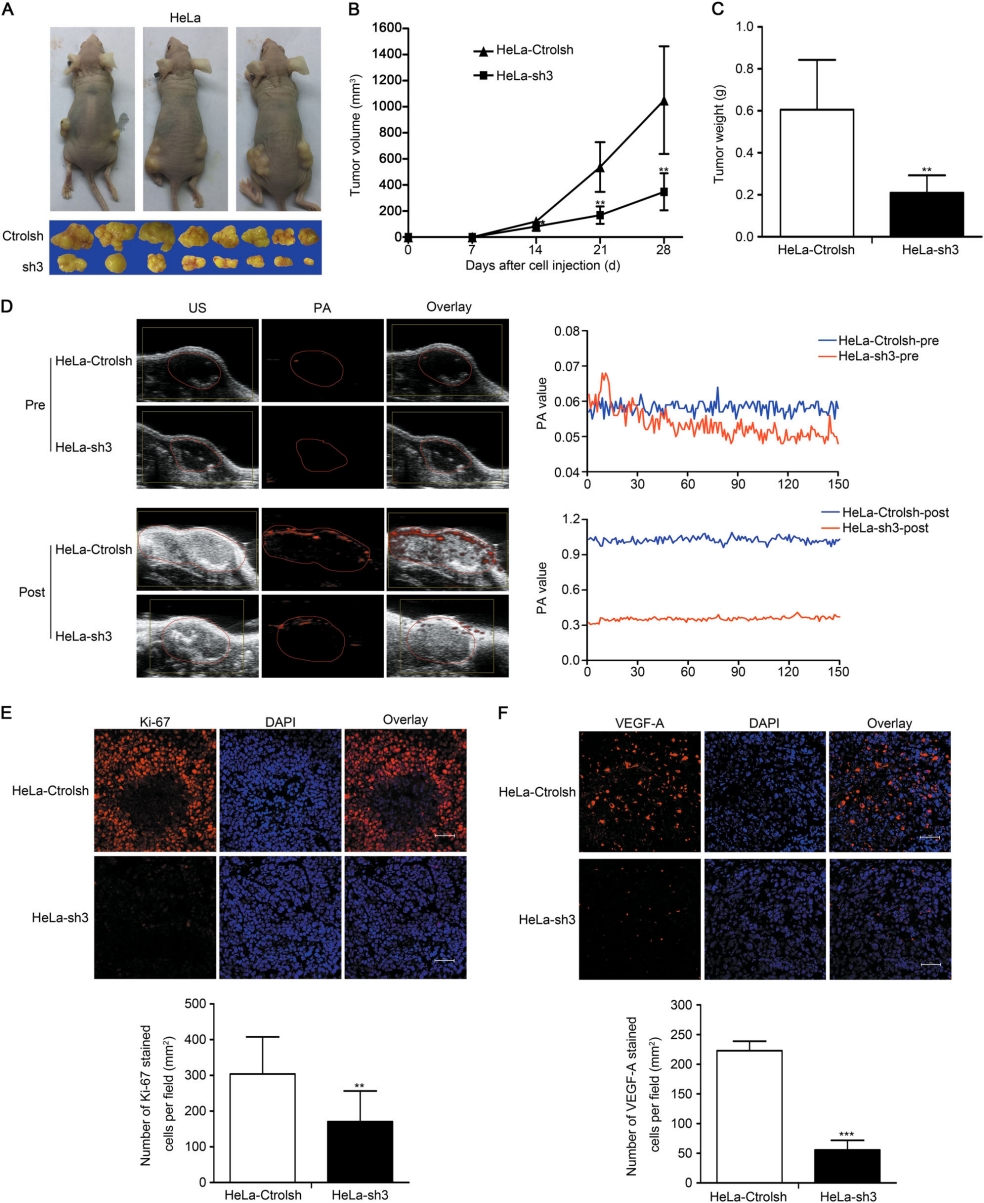
Knockdown of NF90 reduces tumorigenesis and angiogenesis in vivo. 5×10^6^ of HeLa-Ctrolsh cells or HeLa-sh3 cells were subcutaneously injected into the left or right flank of BALB/c nude mice, respectively. **a** Images of cervical cancer xenografts. Top, representative images of cervical cancer tumors; bottom, all of the resected tumors from individual mice, *n* = 8. **b** The volume of the tumors. Points, mean (*n* = 8), bars, SD. *P < 0.05, **P < 0.01. **c** The weight of tumors. Columns, mean (*n* = 8), bars, SD. **P < 0.01. **d** Representative PA images and PA values in tumors of two groups. PA images and PA values were captured before and after 4 h of the injection of PEG-AuNR through tail vein. Tumors are marked with red solid lines; *n* = 8. **e** The Ki-67 protein expression of tumor tissues was evidenced by immunofluorescence (top, immunofluorescence; bottom, quantified results). Red, Ki-67 staining; blue, nuclei stained with DAPI. Magnification, ×400; scale bars = 50 µm. **f** Immunofluorescence analysis of VEGF-A protein expression in the tumor tissues (top, immunofluorescence; bottom, quantified results). Red, VEGF-A; blue, nuclei stained with DAPI. Magnification, ×400; scale bars = 50 µm. Images shown in **e** and **f** are representative of 10 random fields of 5 independent experiments. Column, mean (*n* = 5); bars, SD. **P < 0.01, ***P < 0.001. Results were analyzed using paired *t*-test

The findings that NF90 promotes VEGF-A expression and angiogenesis of cervical cancer cells in vitro prompted us to test whether NF90 deficiency may affect ectopic tumor growth and angiogenesis in tumor xenograft assays. Cervical cancer carcinoma (HeLa) with stable expression of either shRNA control (ctrolsh) or NF90 shRNA3 (sh3) were established. The 4–6-week-old BALB/c nude mice received subcutaneous implantations of Hela-Ctrolsh in the left, and Hela-sh3 in the right flank. Tumor formation was observed and tumor weight was measured in these groups. As predicted, compared with ctrolsh mice, the tumor size was considerably reduced in NF90 knockout mice (Fig. 6a). The average volume and weight of tumors in NF90 knockdown mice was significantly lower than that in HeLa-ctrolsh mice (Fig. 6b, c). In addition, immunofluorescence analysis of the expression of Ki-67, a tumor cell proliferation marker, revealed that the expression of Ki-67 in cervical cancer tumors of HeLa-ctrolsh mice was higher than that of NF90 knockdown mice (Fig. 6e). These observations clearly demonstrated that downregulation of NF90 inhibited tumorigenesis by repressing cervical cancer cell proliferation in vivo.

Photoacoustic imaging (PA) also been used to detect the microvessel process in mice. The PA values of NF90 knockdown mice were lower than that of HeLa-Ctrolsh mice (Fig. 6d). Significantly, compared with HeLa-Ctrolsh mice, the immunofluorescence analysis indicated that the levels of VEGF-A expression were significantly reduced in NF90 knockdown mice (Fig. 6f). These findings further determined that NF90 depletion compromises tumorigenesis and angiogenesis of cervical cancer tumors.

## Discussion

In this study, we find the upregulation of NF90 and NF110 in cervical cancer specimens. And further provide novel evidence regarding the enhancement of NF90 on the expression and secretion of VEGF-A in cervical cancer, which plays a cardinal role in regulating hypoxia-induced tumor angiogenesis. In particular, NF90 is demonstrated stimulating the expression and secretion of VEGF-A through the PI3K/Akt transduction pathway under hypoxic stress. As a biological counterpart, the silence of NF90 inhibits tubule-like structure formation in HUVECs. Further corroborating these findings, we demonstrated that the depletion of NF90 reduces the neonatal vascular process and tumor capacity of cervical cancer cell lines in nude mice.

The location of NF90 and NF110, its larger splice variant, is not the same. One portion of NF90 is in the nucleus via dsRBM-mediated RNA interactions, while the other establishes more significant ribonucleoprotein contacts. It appears that NF110 is exclusively nuclear^46^. NF45 is combines with the N-terminal and C-terminal of NF90 to form NF90/NF45 heterodimer core complex, and alters the RNA-binding specificity of NF90^4^. The NF90/NF45 complex may exert different functions from monomeric NF90 in the cells^47^. Previous report showed that repression of either NF90 or its regulatory subunit NF45, but not NF110, leads to the retardation of cervical cancer cell growth and formation of giant multinucleated cells^4^. Further, suppression of NF90 delays cell cycle progress and cell growth in human hepatocellular carcinoma (HCC) cell lines through binding to the 3′-UTR of Cyclin E1 mRNA, and reduces the sensitivity of HCC cells to the roscovitine, a CDK inhibitor that is applied in II experiments^18^. Overexpression of NF90 is reported in nasopharyngeal carcinoma, non-small-cell lung cancer, ovarian cancer, and hepatocellular carcinoma^3,18,48,49^. Elevated NF90 staining correlates with the higher pathological grading, and the nuclear localization highlights its role as transcription factor in invasive breast cancer cells^27^. In our study, we find NF90 is upregulated in cervical cancer specimens. But the correlation of NF90 expression with the grading and prognosis of cervical cancer has not been determined, with the limited number of cases. A large number of cases and long-term follow-up are needed to better understand the potential functions of NF90 in cervical cancer. Meanwhile, NF45 was previously recognized as just a regulatory subunit of complexes with NF90/NF110, and regulated the stabilization of complexes^4^. By now, researchers find the NF45 not only participates in cell cycle regulation and cell proliferation, but also acts as a novel therapeutic target for malignant glioma or esophageal squamous cell carcinoma^50,51^. Further investigation of NF90/NF45 and NF110/NF45 complex for better understanding of the molecular mechanism of tumorigenesis and the identification of potential biomarkers of cervical cancer are demanded.

Hypoxic stress of solid tumors urges cells toward aggressive biological properties with the help of anti-apoptotic, proliferative, and angiogenic factors^30^. HIF-1α is a key transcriptional factor of cellular adaptation to hypoxic stress and activating genes involved in cell survival, angiogenesis, migration, and invasion^52,53^. Hypoxic stress-activated HIF-1α acts as a survival factor by modulating various signaling pathways^44,54–56^ and triggering gene transcription, including VEGF-A^57^. The expression of VEGF-A does not always depend on HIF-1α transcription, although VEGF-A is the target gene of HIF-1α. Post-transcriptional mRNA stability and mRNA transport mechanisms are demanded for the VEGF-A expression under hypoxia. In addition, AU-rich elements (AREs) in the 3′-UTR stem-loop hypoxia stability region are most important for the VEGF-A mRNA stability under hypoxia^58^. On the other hand, NF90 recognizes specific bases in dsRNA through an ADAR2-like binding mode^59^. The DPBP76/NF90 isoform binds to the VEGF-A 3'-UTR, facilitates hypoxia-induced VEGF-A mRNA levels and protein translation without affecting HIF-1α transcription, and indicates that DPBP76/NF90 functions as a post-transcriptional regulator^16^. Analogous, NF90/VEGF-A signaling axis promoted the angiogenesis, tumor growth, and lung metastasis of colorectal cancer^60^. In the present study, we further revealed that NF90 may be a regulator of VEGF-A expression through HIF-1α-dependent way under hypoxia in cervical cancer. These results suggested that NF90 may be a new target for anti-angiogenesis, and the specific mechanism of the NF90 influences on the expression of HIF-1α and VEGF-A is on the way.

Altogether, our findings summarize that NF90 is upregulated in cervical cancer specimens, and the participation of NF90 is integral in the regulation of pivotal genes involved in angiogenesis and metastasis in human cervical cancer cells exposed to hypoxia. Moreover, further studies about the interplay between NF90 and HIF-1α-induced VEGF-A expression are forward looking. The cooperation between NF90, HIF-1α, and VEGF-A on the molecular mechanism driving the biological response to hypoxic condition and the role in diverse pathophysiological conditions also need to be further elucidated.

## Materials and methods

### Human tumor specimens

Fourteen paired cervical cancer specimens and the adjacent normal cervical tissues were collected at primary surgery prior to chemotherapy or radiotherapy in the Department of Obstetrics and Gynecology of the Second Affiliated Hospital of Chongqing Medical University (Chongqing, China). All samples were snap-frozen in liquid nitrogen and stored at −80 °C until further analysis. And they were evaluated by pathologists and stratified as squamous cell carcinoma (*n* = 8), adenocarcinoma (*n* = 4), or adenosquamous carcinoma (*n* = 2). The details of patients were shown in Supplementary Table 1.

### Analysis of mRNA and protein expressions of NF90 and NF110 in human cervical cancer

The protein expressions of NF90 and NF110 in human cervical squamous cell carcinoma and adenocarcinoma tissues were determined from the Human Protein Atlas (www.proteinatlas.org). The ILF3 gene expression of human cervical cancer was determined through analysis of Pyeon Multi-cancer database, which is available through Oncomine (www.oncomine.org). High and low groups were defined as above and below the mean, respectively.

### Cell lines and hypoxic stimulation

The human cervical cancer cell lines HeLa and SiHa were cultured in DMEM supplemented with 10% fetal bovine serum (FBS) and 100 μg/ml penicillin/streptomycin. HUVECs were seeded on 1% gelatine-coated flasks and cultured in HUVEC growth medium, composed of M199 supplemented with 20% FBS, 30 μg/ml endothelial cell growth supplement from bovine neural tissue, and 10 U/ml heparin.

For hypoxic stimulation, cells were starved overnight with serum-free medium, and were treated with CoCl_2_ (150 μM, Sigma-Aldrich, USA) or cultured in the presence of low-oxygen tension (2% O_2_) in a Forma incubator (Thermo Fisher Scientific, USA) for indicated durations.

### Western blotting

Cell extracts were prepared following the manufacturer’s instructions. Protein samples (30–60 μg total protein per lane) were separated by SDS-PAGE and then transferred onto PVDF membranes (Millipore, USA). Blots were blocked with 5% non-fat milk or 5% BSA in 0.1% Tween-TBS and incubated with indicated antibodies at 4 °C overnight, followed by incubation with appropriate horseradish peroxidase-conjugated secondary antibodies (Abbkine, USA). The levels of proteins and phosphor proteins were revealed using WesternBright^TM^ ECL System (APGBio, China). Antibodies were against NF90 (ILF3, Abcam, USA), NF45 (ILF2, Abcam), VEGF-A (Abcam), HIF-1α (Novus, USA), Akt (CST, USA), p-Akt (Abcam), β-actin (EarthOx, USA), and β-tubulin (EarthOx). The PI3K inhibitors LY294002 and Wortmannin were purchased from Cell Signaling Technology (CST, USA), and Akt inhibitor MK-2206 was purchased from Selleck (USA).

### Confocal microscopy

Forty percent confluent cultured HeLa cells grown on coverslips were serum-deprived for overnight and treated with CoCl_2_ for 12 h. Then coverslips were fixed using 4% paraformaldehyde, permeated with 0.1% Triton X-100, and incubated with blocking buffer (10% goat serum in PBS) at room temperature for 1 h. After PBS wash, coverslips were incubated with VEGF-A (Abcam) or HIF-1α (Novus) antibody overnight at 4 °C, and then incubated with Dylight 549 (Abbkine, USA) for 1 h at room temperature. 4′, 6-diamidino-2-phenylindole (DAPI, Beyotime, China) was used for nuclear staining. Nikon A1^+^ R Real-Time Full-Spectrum Double Sweep Laser Scanning Confocal Microscope supported by quantification and image processing software NIS-Elements 4.3 (Nikon, Japan) was used for experiment evaluation.

Fresh resected tumor tissues were embedded in O.C.T. compound (Sakura, USA) and sliced into consecutive sections with a thickness of 10 μm utilizing a vibrating microtome (Leica CM1950, Nussloch, Germany). The slices were incubated with VEGF-A (Abcam) or Ki-67 (Abcam) antibody overnight at 4 °C

### ShRNAs/siRNAs

For knockdown experiments, NF90 shRNAs were purchased from Hanbio Biotechnology (China), and were produced by cotransfection of 293T cells with recombinant lentiviral shRNA vector (pHBLV-U6-ZsGreen-PGK-Puro) containing the NF90 target sequences (NF90/sh1-3) and the negative control (named ctrolsh). ShRNA/siRNA sequences are shown in Supplementary Table 2. Stable lentiviral transfected HeLa and SiHa cells were selected using puromycin, adding the minimum concentration required to kill untransfected cells. The efficiency and specificity of knockdown were confirmed by green fluorescence, PCR, and western blotting.

For NF90 overexpression, the NF90 cDNA was subcloned into the pHBLV-CMVIE-ZsGreen-Puro vector containing a Flag tag and puromycin resistance gene for the establishment of stable transfectants (named NF90). Overexpression control cells were generated in parallel (named control).

### Real-time quantitative PCR

Total RNA was extracted from the cell cultures using Trizol reagent (Takara, Japan) according to the manufacturer’s protocol. qRT-PCR analysis was conducted using a SYBR^®^ Premix Ex Taq^TM^ II (Takara, Japan). Properly diluted cDNA was used in a 10 μl qRT-PCR in triplicate for every gene. The cycle parameters were 95.0 °C for a 30s hot start, 40 cycles of 95.0 °C for 5 s, and 60.0 °C for 30 s. The relative gene expression levels were calculated using the 2ΔΔCT method^61^. Gene expression levels were normalized to the GAPDH housekeeping gene. All data were expressed relative to values obtained for negative control cells (value = 1). The primers for each gene are shown in Supplementary Table 3. HIF-1α inhibitor PX-478 (Selleck, USA) was used to investigate whether NF90 may regulate the expression of VEGF-A in HIF-1α-dependent way.

### Conditioned medium (CM)

HeLa cells with NF90 stable knockdown or overexpression were cultured in regular growth medium (DMEM + 10% FBS + 1% penicillin/streptomycin) to 80% confluence. Then, cells were washed twice with PBS and starved overnight with serumfree medium (DMEM). Subsequently, cells were stimulated with CoCl_2_ (150 μM) for 12 h. Thereafter, the culture supernatants were collected, centrifuged at 16,000×*g* for five minutes to remove cell debris and stored at −80 °C until use. In the series of experiments, HeLa cells were pre-treated for 30 min with inhibitors including LY294002, Wortmannin, and MK-2206, and then cultured with CoCl_2_ (150 μM) for 12 h to prevent signaling via the NF90.

### Enzyme-linked immunosorbent assay  (ELISA)

The concentrations of secretory VEGF-A protein in the CM from untreated and treated cells were detected using human VEGF ELISA Development kit (Peprotech, USA) according to the manufacturer’s instruction. The results were normalized to 2 × 10^6^ cell number. All experiments were repeated in triplicate.

### Tube formation assay

80 Percent confluent HUVECs were starved overnight. Chilled liquid Matrigel^®^ (Corning, USA) was dispensed into pre-chilled (on ice) 96-well plate (50 µl per well) and allowed to solidify for 1 h at 37 °C. Starved HUVECs were resuspended in CM and seeded on Matrigel^®^ at 10,000 cells per well and incubated at 37 °C. The formation of capillary-like structures was captured starting from 2 h after cell seeding by microscope (DM6000B, Leica, Germany). The tubule crosses were quantified by using the software Image-Pro Plus 6.0 (Media Cybernetics, USA). All experiments were repeated in triplicate.

### Cell migration assay

Migration activity of HUVECs was assessed using a transwell assay (BD Biosciences, USA, pore size, 8 µm). Briefly, 10,000 cells were seeded in the upper chamber in 100 µl of 10% FBS complete medium. In the lower chamber in 300 µl containing 150 µl 20% FBS complete medium and 150 µl CM. After 24 h of incubation at 37 °C, cells were fixed with 4% formaldehyde and subsequently stained with 0.1% crystal violet. Cells on the upper side of the filters were removed, and washed with PBS. Migrated cells were examined and counted under a microscope (DM6000B, Leica, Germany). Each culture condition was conducted in triplicate.

### Angiogenesis and tumor growth analysis in vivo

BALB/c 4–6-week-old female nude mice (nu/nu) were purchased from Beijing HFK Bio-science Co., Ltd. (Beijing, China, license number, SCXK-20140004), and were kept in institutional pathogen-free facilities of Chongqing Medical University. Protocols to work with these animals were approved by the Chongqing Medical University Institutional Animal Care and Use Committee. Briefly, 5 × 10^6^ HeLa cells transfected with negative control or NF90 shRNA3 were subcutaneously injected into the left or right flank of nude mice, respectively. Approximately seven days after injection palpable tumors became detectable and were measured weekly. The mice were sacrificed when the tumors of negative control reached a volume of ~1500 mm^3^. Tumors were extracted, measured, divided, and stored at −80 °C.

To better observe the neovascularization of tumors, PA tomography was performed using the Vevo^®^ LAZR Imaging System (VisualSonics. Canada) in vivo. The procedure was performed as previously mentioned^62^. In brief, the ultrasound (US) and PA images of the tumors were captured at prior to, immediately, 1, 2, 4, 6, and 24 h after mice had been injected 100 µl polyethylene glycol-modified gold nanorods (PEG-AuNR, NanoSeedz, China) from the tail vein^63^. Two hundred images per tumor were captured continuously for analysis.

### Statistical analysis

Statistical analysis was performed using SPSS 23.0. Statistical, and differences among groups were analyzed by paired Student’s *t* test or one-way ANOVA. All data are presented as the mean ± standard deviation (SD). P < 0.05 was considered as significant.

## Electronic supplementary material

Supplementary Table 1. Clinicopathological features of the cervical cancer cases

Supplementary Table2. shRNA/siRNA sequences

Supplementary Table 3. Primers for PCR

Supplementary Figure1. Hypoxia induces the expression of HIF-1α, VEGF-A and NF90 in cervical cancer cells

Supplementary Figure2. The mRNA expressions of NF110, NF90 and NF45 after transfected with NF90 shRNA

Supplementary Figure Legends
