# Supplementary material for: Nuclear factor 90 promotes angiogenesis by regulating HIF-1α/VEGF-A expression through the PI3K/Akt signaling pathway in human cervical cancer
Source: Cell Death Dis. 2018 Feb 15;9(3):276. doi: 10.1038/s41419-018-0334-2 (PMC5833414; doi:10.1038/s41419-018-0334-2)
Supplement: Supplementary file 1 — Supplementary Table 1. Clinicopathological features of the cervical cancer cases [file 41419_2018_334_MOESM1_ESM.docx]

**Supplementary Table 1. Clinicopathological features of the cervical cancer cases.**

| Clinicopathological features | Cases |
| --- | --- |
| Age (years) |  |
| ≤ 50 | 11 |
| > 50 | 3 |
| Pathological type |  |
| Squamous cell carcinoma | 8 |
| Adenocarcinoma | 4 |
| Adenosquamous carcinoma | 2 |
| HPV infection |  |
| HPV 16 (+) | 6 |
| HPV 18 (+) | 3 |
| Other types | 1 |
| FIGO stage |  |
| I | 7 |
| II | 7 |
| Histological differentiation |  |
| Well | 5 |
| Moderate | 8 |
| Poor | 1 |
| Number of tumor nodules |  |
| 1­ | 1 |
| ≥2 | 3 |
| Vascular invasion | 3 |
