# Supplementary material for: Nuclear factor 90 promotes angiogenesis by regulating HIF-1α/VEGF-A expression through the PI3K/Akt signaling pathway in human cervical cancer
Source: Cell Death Dis. 2018 Feb 15;9(3):276. doi: 10.1038/s41419-018-0334-2 (PMC5833414; doi:10.1038/s41419-018-0334-2)
Supplement: Supplementary file 2 — Supplementary Table2. shRNA/siRNA sequences [file 41419_2018_334_MOESM2_ESM.docx]

**Supplementary Table2. shRNA/siRNA sequences**

| shRNA | Sequence (5’-3’) |
| --- | --- |
| NF90 negative control (Ctrolsh) | TTCTCCGAACGTGTCACGTAA |
| NF90 shRNA1(sh1) | CAGACTGCTACGGCTATCA |
| NF90 shRNA2(sh2) | CTCAAAGCTGTGTCCGACTGGATA |
| NF90 shRNA3(sh3) | CCACTGATGCTATTGGGCATCTAGA |
