# Supplementary material for: Nuclear factor 90 promotes angiogenesis by regulating HIF-1α/VEGF-A expression through the PI3K/Akt signaling pathway in human cervical cancer
Source: Cell Death Dis. 2018 Feb 15;9(3):276. doi: 10.1038/s41419-018-0334-2 (PMC5833414; doi:10.1038/s41419-018-0334-2)
Supplement: Supplementary file 3 — Supplementary Table 3. Primers for PCR [file 41419_2018_334_MOESM3_ESM.docx]

**Supplementary Table 3. Primers for PCR**

| Gene |  | Sequence (5’-3’) |
| --- | --- | --- |
| NF90 | F | ATGCGTCCAATGCGAATTTTTG |
|  | R | CTTTTAGACGCTCTAGGAAGACCCA |
| NF110 | F | GGCTCCTACTACCAAGGTGACAACT |
|  | R | TTATAGCCTTTCTGCTTGCCCT |
| NF45 | F | CACCCTGGATCCTTGACCTA |
|  | R | AACAGTCCTGCAGCCAGAAT |
| VEGF-A | F | CGCAAGAAATCCCGGTATAA |
|  | R | AAATGCTTTCTCCGCTCTGA |
| GAPDH | F | AGAAGGCTGGGGCTCATTTG |
|  | R | AGGGGCCATCCACAGTCTTC |
