# Supplementary figures and images for: Nuclear factor 90 promotes angiogenesis by regulating HIF-1α/VEGF-A expression through the PI3K/Akt signaling pathway in human cervical cancer
Source: Cell Death Dis. 2018 Feb 15;9(3):276. doi: 10.1038/s41419-018-0334-2 (PMC5833414; doi:10.1038/s41419-018-0334-2)

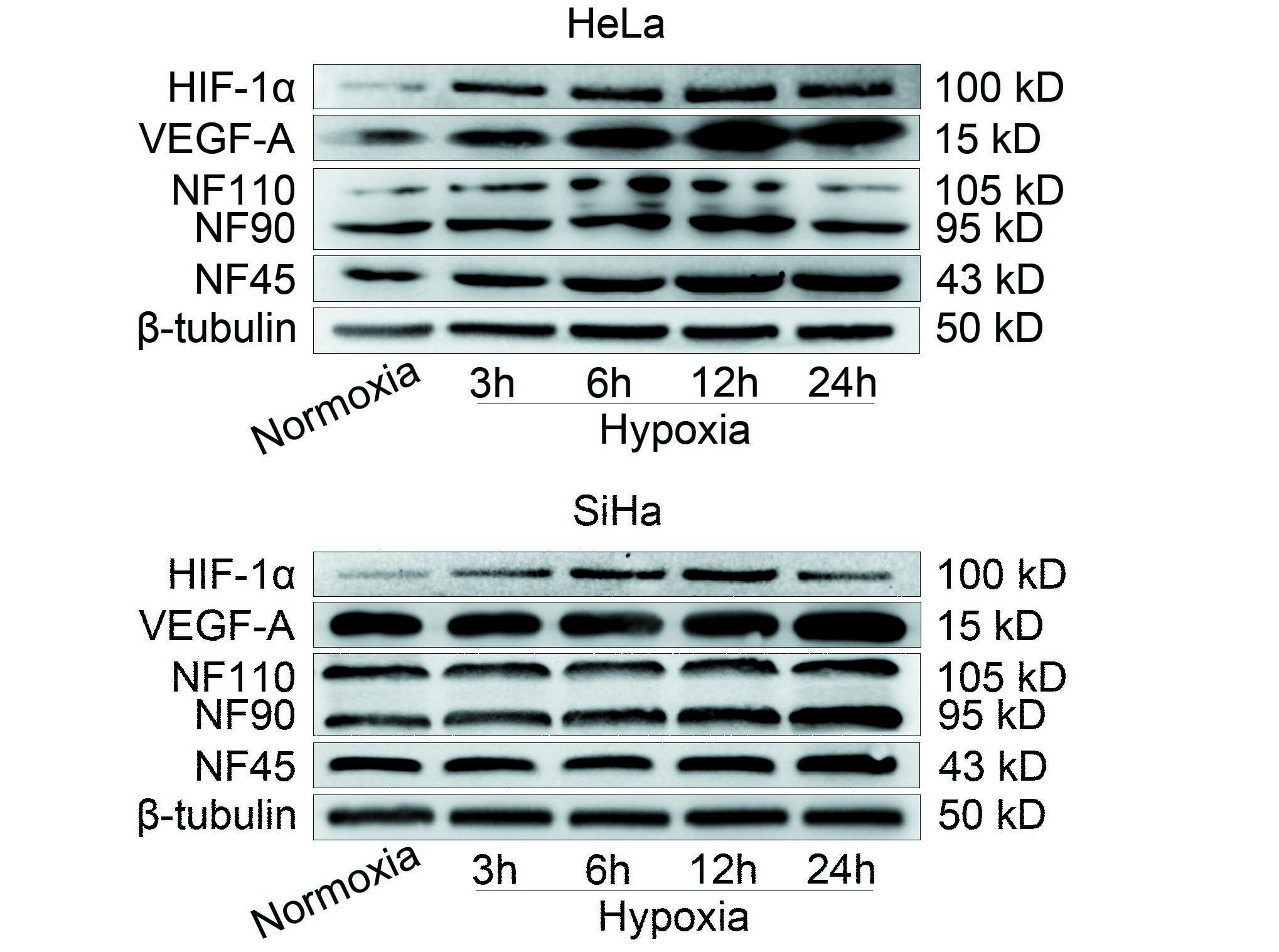

Supplement: Supplementary file 4 — Supplementary Figure1. Hypoxia induces the expression of HIF-1α, VEGF-A and NF90 in cervical cancer cells [file 41419_2018_334_MOESM4_ESM.jpg]

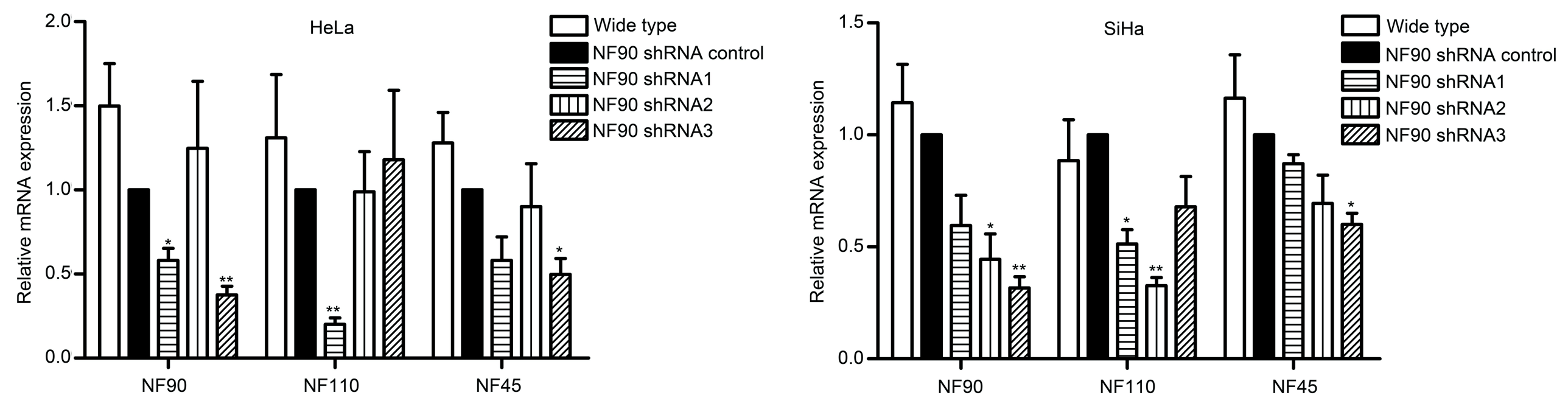

Supplement: Supplementary file 5 — Supplementary Figure2. The mRNA expressions of NF110, NF90 and NF45 after transfected with NF90 shRNA [file 41419_2018_334_MOESM5_ESM.jpg]
