# Supplementary material for: Nuclear factor 90 promotes angiogenesis by regulating HIF-1α/VEGF-A expression through the PI3K/Akt signaling pathway in human cervical cancer
Source: Cell Death Dis. 2018 Feb 15;9(3):276. doi: 10.1038/s41419-018-0334-2 (PMC5833414; doi:10.1038/s41419-018-0334-2)
Supplement: Supplementary file 6 — Supplementary Figure Legends [file 41419_2018_334_MOESM6_ESM.docx]

**Supplementary Figure Legends**

**Supplementary Figure 1. Hypoxia induces the expression of HIF-1α, VEGF-A and NF90 in cervical cancer cells.** HeLa and SiHa cell lines were incubated under normal (21% O_2_) or hypoxic oxygen tension (2% O_2_) from 3 h to 24 h after starved overnight. The protein expression of HIF-1α, VEGF-A, NF90, NF45 and NF110 were determined by western blotting. All experiments were repeated in triplicate.

**Supplementary Figure 2. The mRNA expressions of NF110, NF90 and NF45 after transfected with NF90 shRNA.** Cervical cancer cells (1×10^6^) were seeded in 6-well plates and cultured overnight. Cells were transfected with lentivirus containing shRNA directed against NF90 (1-3) or negative control (Ctrolsh). After selected with puromycin for 14 days, cells were extracted using RNAiso Plus. The relative mRNA expression of NF90, NF110 and NF45 was evidenced by qRT-PCR, and GAPDH as control. Column, mean (n=3). *P <0.05, **P <0.01, compared with Ctrolsh.
